# Harnessing Radiation for Nanotechnology: A Comprehensive Review of Techniques, Innovations, and Application

**DOI:** 10.3390/nano14242051

**Published:** 2024-12-21

**Authors:** Mobinul Islam, Md. Shahriar Ahmed, Sua Yun, Hae-Yong Kim, Kyung-Wan Nam

**Affiliations:** Department of Energy and Materials Engineering, Dongguk University, Seoul 04620, Republic of Korea; mobin85@dongguk.edu (M.I.); shahriar.emcl@dgu.ac.kr (M.S.A.);

**Keywords:** nanomaterial, electron beam irradiation, gamma radiation, X-ray beam, application of nanomaterials, metallic nanoparticle

## Abstract

Nanomaterial properties such as size, structure, and composition can be controlled by manipulating radiation, such as gamma rays, X-rays, and electron beams. This control allows scientists to create materials with desired properties that can be used in a wide range of applications, from electronics to medicine. This use of radiation for nanotechnology is revolutionizing the way we design and manufacture materials. Additionally, radiation-induced nanomaterials are more cost effective and energy efficient. This technology is also having a positive impact on the environment, as materials are being produced with fewer emissions, less energy, and less waste. This cutting-edge technology is opening up new possibilities and has become an attractive option for many industries, from medical advancements to energy storage. It is also helping to make the world a better place by reducing our carbon footprint and preserving natural resources. This review aims to meticulously point out the synthesis approach and highlights significant progress in generating radiation-induced nanomaterials with tunable and complex morphologies. This comprehensive review article is essential for researchers to design innovative materials for advancements in health care, electronics, energy storage, and environmental remediation.

## 1. Introduction

Nanomaterials have emerged as a pivotal class of materials with transformative potential across multiple industries due to their unique properties at the nanoscale. These materials, characterized by dimensions typically between 1 and 100 nanometers [1,2], exhibit distinctive mechanical, optical, electrical, and chemical properties that are not present in their bulk counterparts. Nanomaterials are increasingly important in today’s world due to their unique and versatile properties that can be harnessed across a wide range of applications, driving innovation and efficiency in various fields. The increased surface area to volume ratio at this scale enhances their reactivity, making them ideal for applications in catalysis, electronics, and medicine [3]. Their high surface area to volume ratio enhances chemical reactivity, making them valuable in catalysis and environmental applications such as pollution control and water purification. In medicine, nanomaterials are revolutionizing drug delivery systems, allowing for targeted therapies that minimize side effects and improve efficacy, as well as enhancing diagnostic imaging techniques [4]. In the realm of electronics, nanomaterials enable the miniaturization of components, leading to the development of faster, smaller, and more energy-efficient devices. Additionally, they contribute to advancements in energy storage and conversion, such as in the development of high-capacity batteries and efficient solar cells. The ability to tailor the properties of nanomaterials for specific needs also offers significant potential in the development of strong yet lightweight materials for use in transportation and construction. Overall, nanomaterials are a driving force in advancing technology and improving the quality of life, addressing global challenges, and contributing to sustainable development [5].

In other events, radioactivity, nuclear reactors, Hiroshima and Nagasaki, and Chernobyl are common images that people think about when they think about radiation. However, radiation also exists in many different forms, including visible light, infrared light, solar radiation, UV light that produces a suntan, and radio and television signals. The word radiation refers to the way that energy is released from a nucleus in the form of waves or particles. This energy can travel through space, and when it interacts with matter, it can cause changes in the matter. These changes can be in the form of heat, light, or other forms of energy. Radiation is a vital component of life on Earth because it surrounds every living organism. It is an important part of photosynthesis, the process by which plants use light to convert carbon dioxide and water into glucose and oxygen. As both plants and animals use radiation to send and receive signals, it is also crucial for interspecies communication. Radiation is also an important part of the human body, as it is used in medical imaging and cancer treatments. Radiation is used predominately to detect nuclear waste and measure long-distance objects. It is important for the weather forecasting sector, as it is used to detect changes in air pressure and temperature, volcanic activity, and earthquakes. The nanomaterials in driving technological advancements stem from their versatile properties and superior performance compared to their bulk counterparts. Traditionally, nanoparticles have been synthesized by reducing metal ions into neutral particles using harmful reducing agents. However, recent efforts have focused on developing green radiation technologies that leverage radiation sources instead of hazardous chemicals for nanoparticle production. This shift aims to enhance the safety and environmental sustainability of synthesis methods while maintaining the effectiveness of the resulting nanoparticles [6]. The synthesis of nanomaterials using radiation technology is an innovative and impactful approach that leverages various forms of radiation, including gamma rays, electron beams, and ultraviolet (UV) light [7,8,9,10,11,12], to produce nanoparticles with well-defined characteristics. This method offers exceptional precision in controlling reaction conditions, allowing nanoparticles that exhibit uniform size and shape, essential factors for their performance in specific applications. The synthesis process typically begins with the preparation of a precursor solution containing metal salts or organic molecules, which are then subjected to irradiation. This irradiation effectively generates free radicals, instigating chemical reactions that lead to the formation of nanomaterials [13,14]. One of the most significant advantages of using radiation technology for synthesis is the high purity of the resulting nanoparticles, as the technique often eliminates the need for additional chemical reagents, thereby reducing the risk of contamination. Moreover, radiation methods are more environmentally friendly, generating less chemical waste and minimizing the reliance on toxic substances compared to traditional synthesis methods, which aligns with sustainable practices in materials science [15,16,17]. The energy efficiency of radiation-based processes further enhances their appeal, as they generally require less energy expenditure than conventional thermal methods. The resulting nanomaterials find valuable applications across diverse fields, including biomedicine, where they are used for targeted drug delivery and advanced imaging techniques, as well as in electronics, enabling the miniaturization of components for faster, more efficient devices. Additionally, these nanoparticles play a vital role in catalysis, enhancing reaction rates and selectivity in chemical processes. Despite the numerous advantages, challenges such as the scalability of the process for industrial applications and the need for consistent reproducibility must be addressed. Continued research and innovation in radiation synthesis techniques holds the promise of expanding the capabilities and applications of nanomaterials [1,18,19], making them an important area of focus in the advancement of nanotechnology and its integration into various industries. Gamma rays, ultraviolet (UV) light, and electron beam irradiation play crucial roles in the synthesis of nanomaterials [10,11], each providing unique mechanisms and advantages that enhance the formation and properties of nanoparticles. Gamma irradiation, known for its high energy and penetrating power, generates free radicals in precursor solutions, initiating chemical reactions that reduce metal ions into high-purity nanoparticles, essential for applications in catalysis, electronics, and biomedicine. Meanwhile, UV irradiation excites atoms or molecules in precursor solutions, promoting environmentally friendly reactions without the use of toxic chemicals, effective for synthesizing metal nanoparticles and semiconductor nanostructures with controlled morphology and composition. Electron beam irradiation directs high-energy electrons into substrates or solutions, allowing precise control over synthesis parameters and facilitating various reactions, which results in the generation of consistent nanoparticles [20]. Together, these modern radiation techniques offer significant advantages over conventional methods, leading to enhanced functionalities and diverse applications of nanomaterials in multiple industries.

**Scheme 1:**
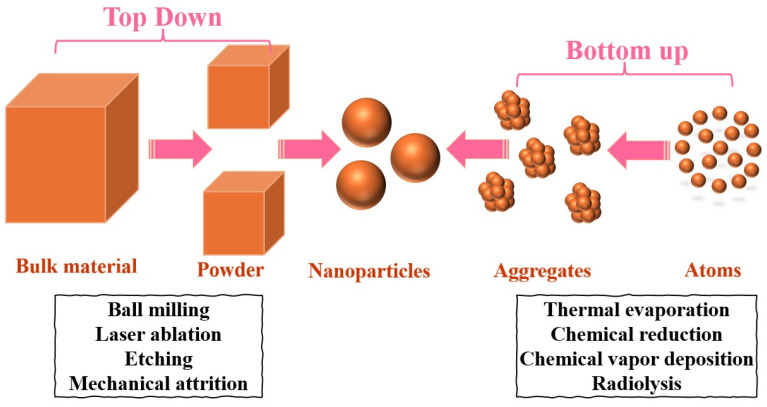
The scheme illustrates top-down and bottom-up approaches for the synthesis of nanostructures.

The synthesis of nanomaterials, while offering significant advantages, presents several challenges associated with both top-down and bottom-up approaches [21,22,23]. In top-down techniques like mechanical milling and lithography, a key challenge is achieving precise control over the size, shape, and distribution of nanoparticles (as shown in Scheme 1). Variability in these parameters can critically affect the performance of the materials in specific applications. Moreover, these methods often require complex, expensive equipment and controlled conditions, which can escalate production costs and limit their feasibility for large-scale manufacturing [24]. On the other hand, bottom-up approaches, such as chemical vapor deposition and sol–gel processes, face challenges related to the purity and stability of the synthesized nanomaterials. Contaminants or agglomeration during the synthesis can significantly alter the desired properties and effectiveness of the nanoparticles. Additionally, these methods may involve toxic reagents or byproducts, raising environmental and safety concerns that must be addressed. Both approaches also struggle with reproducibility. Minor variations in synthesis conditions can lead to inconsistent results, complicating the development process and hindering the ability to create standardized materials. Furthermore, there is a continuous need to develop more energy-efficient and sustainable synthesis methods that minimize environmental impact while still yielding high-quality nanomaterials. Addressing these challenges requires ongoing research and innovation to refine existing techniques and explore new synthesis pathways that enhance the capabilities and applications of nanomaterials [8,9,10,11].

**Scheme 2:**
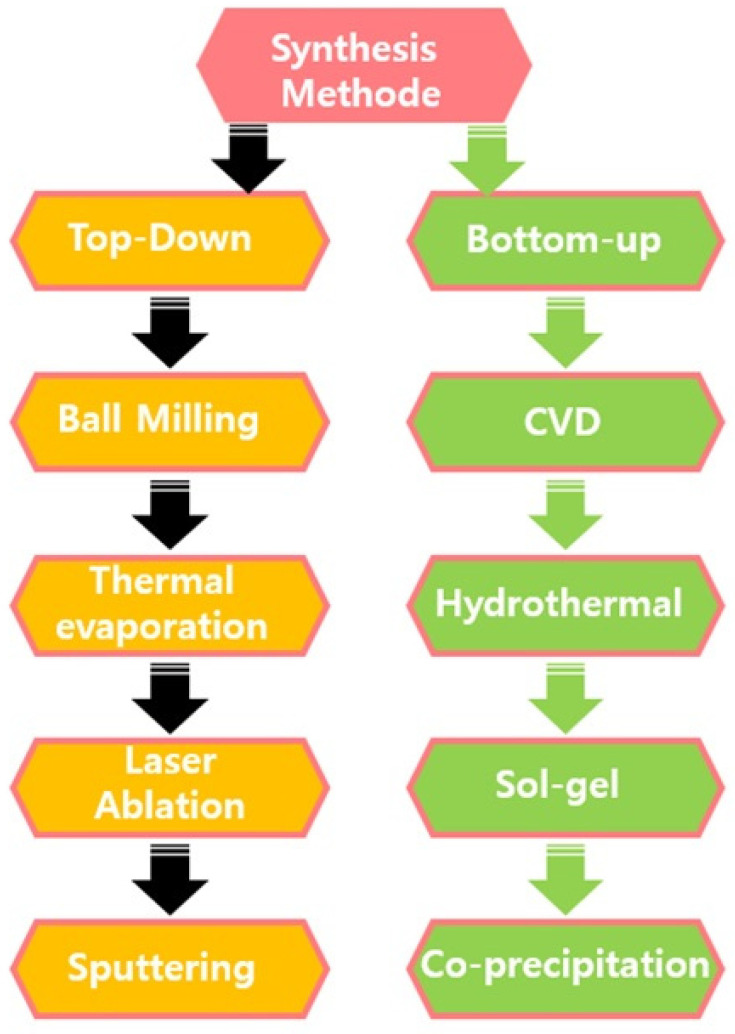
Top-down and bottom-up synthesis techniques for nanomaterials.

Nanotechnology represents a transformative frontier in materials science, offering the potential to revolutionize diverse industries through the development of nanomaterials with tailored properties. At the heart of this transformative power lies the bottom-up synthesis approach, a technique that meticulously constructs nanostructures from fundamental atomic and molecular components. The most common top-down and bottom-up methods are depicted in Scheme 2. Unlike top-down methods, which involve reducing bulk materials to nanoscale dimensions, bottom-up synthesis is distinguished by its ability to achieve unprecedented control over size, shape, and functionalization. This approach encompasses a spectrum of methodologies, including chemical vapor deposition, sol–gel processing, self-assembly, and colloidal techniques. Each method provides unique advantages in precision and scalability, enabling the creation of complex nanostructures optimized for specific applications. For example, chemical vapor deposition allows for the deposition of thin films and nanostructures with precise thickness and composition, while self-assembly leverages the inherent organizational properties of molecules to form well-defined structures. The significance of bottom-up synthesis is particularly evident in fields like electronics, where it aids in the development of nanoscale semiconductors; medicine, through the design of targeted drug delivery systems, diagnostic tools, and therapeutic devices; and energy, by enhancing the efficiency of photovoltaic devices and batteries [25,26]. The ability to engineer materials at the nanoscale not only enhances their physical and chemical properties but also introduces new functionalities that are pivotal for advancing technological innovations. The bottom-up approach, which promotes the growth and aggregation of nanostructures with less mechanical force and energy, often yields amorphous particles with enhanced solubility and uniform size distributions. This method is faster, simpler, and more energy-efficient, making it well-suited for small-scale nanoparticle production [27]. In this section we will provide a comprehensive overview of the bottom-up synthesis techniques for nanomaterials.

### 1.1. Sol–Gel Method

**Figure 1:**
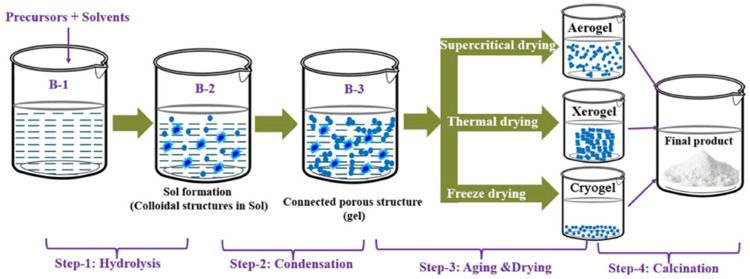
Sol–gel processes from precursor to gel formation, adopted from ref. [28], MDPI.

Among the numerous synthetic methods available to produce high-quality metal oxide nanoparticles and mixed oxide composites, the sol–gel technique is particularly esteemed. This approach enables precise control over the materials’ texture and surface characteristics, particularly using chelating gel precursors (shown in Figure 1) [28]. Variations in experimental conditions and processing parameters during sol–gel synthesis can have a significant impact on the properties of the resulting materials. Therefore, careful consideration of these processing variables is essential to customize the nanoparticles for various applications [29,30]. The sol–gel method is not only simple and cost-effective but also facilitates the achievement of uniform particle sizes, lowers processing temperatures, and promotes the formation of composites and intricate nanostructures [31,32,33].

Various nanoparticles, including metal oxides, mixed oxides, semiconductor nanoparticles, and hybrid organic–inorganic nanoparticles, may be created using the sol–gel process. It is excellent for numerous applications in catalysis, energy storage, sensors, optics, and biomedicine because it enables fine control over particle size, content, and shape. Using isopropanol as a precursor, Ristic et al. synthesized nanocrystalline ZnO powder [34]. Yue et al. synthesized ZnO nanotubes with porous AAO membranes [35].

### 1.2. Chemical Vapor Deposition (CVD)

**Figure 2:**
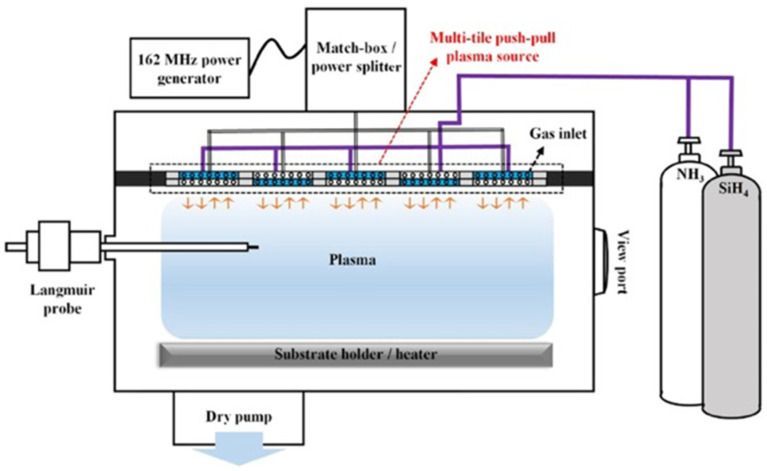
Plasma-enhanced chemical vapor deposition used for synthesis of nanomaterials, adopted from ref. [40], Nature Publication.

The surface can be coated with solid materials created from chemical vapor deposition (CVD), a method that transforms volatile precursors into solid compounds through chemical reactions [36]. This process for depositing powders or films can occur through homogeneous chemical reactions in the gas phase or through heterogeneous reactions on or near the substrate surface [37,38]. Since most CVD processes require energy input due to their endothermic nature, this energy is crucial for the reactions to proceed [39]. Methods such as atomic layer deposition (ALD), along with vapor–liquid and solid growth techniques, provide the ability to control growth processes on a nanoscale, highlighting the critical role of CVD methods in technological advancement (showing in Figure 2) [40]. This significance is further enhanced by ongoing developments in nanotechnology [41]. The CVD synthesis process includes several essential steps: it begins with choosing suitable chemical precursors that will yield the target material. These precursors are then introduced into a reaction chamber in their gaseous state, where they interact with a heated substrate. This interaction initiates a series of surface reactions that lead to the decomposition or reaction of the precursors, forming a solid thin film through nucleation followed by growth. During this process, byproducts are generated and must be eliminated from the chamber to ensure a contamination-free environment. Finally, the properties and quality of the deposited film are assessed through characterization techniques.

In this review, we will focus on the synthesis of nanomaterials using modern radiation technologies, such as gamma rays, and electron beams, offering a comprehensive exploration of these innovative methods. We will highlight the advantages of utilizing radiation technology over traditional top-down and bottom-up approaches, emphasizing the unique benefits this contemporary technique presents. In the final section of this article, we will showcase the diverse applications of nanomaterials in the modern world, illustrating their significant impact across various industries and technologies.

## 2. Synthesis of Nanomaterials by Electron Beam Radiation

Electron beam irradiation is an emerging technique in the synthesis and modification of nanomaterials, providing unparalleled precision and control in nanotechnology applications [42,43,44,45]. This method harnesses high-energy electrons to interact with materials, facilitating reactions such as excitation, ionization, and radiolysis, which are essential for producing and tailoring nanostructures [46]. One of the primary advantages of e-beam irradiation is its capability to induce nucleation and growth of nanoparticles without the need for chemical reagents, leading to environmentally friendly synthesis processes that align with green chemistry principles [47]. In metallic nanoparticle production, e-beam irradiation can precisely control the size and distribution of particles like gold and silver, optimizing their functionality for applications in catalysis and electronics [48,49,50,51,52,53]. In semiconductor fabrication, the technique allows for the creation of quantum dots and nanowires, critical for next-generation electronic devices. Additionally, e-beam irradiation enhances the properties of polymer nanocomposites through cross-linking, making them more suitable for demanding applications in the automotive and aerospace industries.

**Scheme 3:**
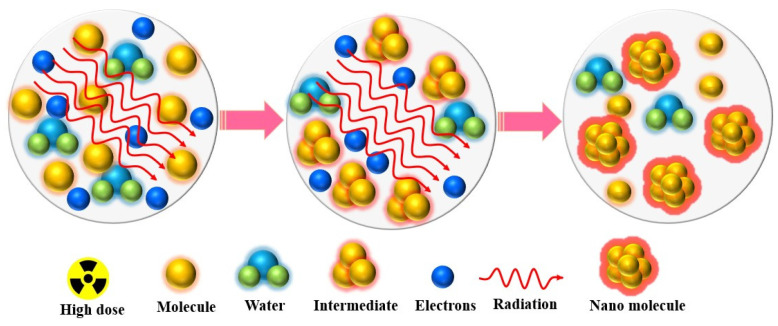
Schematic illustration of nanomaterial synthesis by high irradiation dose.

**Scheme 4:**
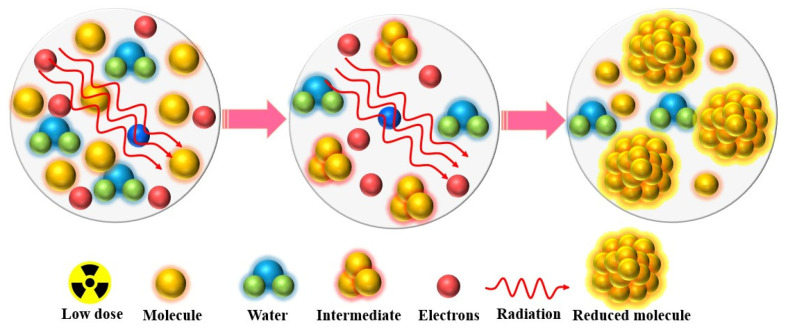
Schematic illustration of nanomaterial synthesis by low irradiation dose.

The irradiation dose and its rate play crucial roles in making the size and shape of nanostructures. The metallic ions reduction to zero-valent atoms depends on the overall reducing classes generated by ionizing radiation, determined by the energy dose deposited in the medium. These reduction reactions occur alongside association and coalescence processes, affecting both free ions and agglomerated ions. The growth and size of nanomaterials are influenced by the rate of reducing species production, which is tied to the energy deposition rate, or dose rate, as depicted in Scheme 3 and Scheme 4. At high dose rates numerous reducing species form quickly, leading to rapid ion reduction and the simultaneous production of numerous free atoms. Each atom acts as a nucleation center, initiating nanoparticle growth, resulting in many small aggregates with a narrow size distribution.

Conversely, at low dose rates fewer reduced species form, causing a slower reduction rate compared to association processes. Initially, few free atoms are produced, and reducing species react more with existing oligomers. With fewer nucleation centers, there are fewer aggregates and particles than at high dose rates, producing larger nanoparticles from the same initial metallic ion concentration.

### 2.1. Electron–Matter Interactions

Recent progress in the understanding of electron–matter interactions, particularly using electron beams in transmission electron microscopy (TEM) and field emission-scanning electron microscopy (FE-SEM), has established these methods as pivotal tools for the remote manipulation of nanomaterials’ functional properties. This technological advancement is characterized by high spatial and temporal resolution, as well as digital control, making them attractive to researchers in the field. The investigation of electron beam interactions with matter is a fundamental aspect of modern science, revealing intricate quantum phenomena associated with electron dynamics. In solid materials, electrons encounter two essential forces: Coulombic repulsion between themselves and attraction toward the atomic nuclei. The interactions between electrons (and/or photons) and atoms at the quantum level yield significant insights into material properties. In TEM, a high-energy electron beam transmits through a sample, forming images that capitalize on the wave-like nature of electrons, similar to techniques used in conventional light microscopy. This capability is critical for analyzing microstructures, characterizing nanoscale materials, and obtaining chemical insights at the atomic scale. However, the high energy associated with the electron beam can induce radiation damage, which may lead to structural alterations and defects that negatively affect the physical and chemical properties of the materials, increasing the risk of failure in practical applications [54,55]. The electron beam generated in TEM and FE-SEM provides a versatile platform for imaging metal nanoparticles, enabling spectroscopic analyses that confirm the presence of specific elements on a localized level. Additionally, e-beam irradiation in TEM not only aids in the fabrication of nanomaterials but also facilitates the investigation of their morphology, structural characteristics, and chemical transformations. These capabilities are essential for developing innovative nanostructures that conventional chemical and physical methods may not be able to produce. Thus, TEM is recognized as a crucial instrument in the field of nanoscale materials engineering. Jiang [56] has compiled a variety of beam damage phenomena associated with oxides in scanning TEM (STEM) and highlighted the shortcomings of currently accepted mechanisms. Similarly, El Mel and Bittencourt [57] have reviewed the processes of solid-to-hollow conversion induced by e-beam irradiation in TEM, citing important examples from the literature. Gonzalez-Martinez et al. [20] conducted a comprehensive review focused on available work related to electron beam-induced synthesis techniques with in situ capabilities, emphasizing the e-beam as a key agent driving the synthesis of nanostructures within the TEM. Demonstrations in recent years have shown that the electron beam in TEM serves as a powerful tool for the fabrication and manipulation of nanostructures, allowing precise control at the nanoscale and even at the individual nanoparticle level. Furthermore, S. Jesse et al. [58] recently reviewed advancements in using focused electron beams to create freestanding nanoscale 3D structures, explore radiolysis and the fabrication potential of liquid precursors, achieve epitaxial crystallization of amorphous oxides with atomic layer precision, and visualize and control the motion of individual dopants within a 3D crystal lattice.

### 2.2. Transition Metal Nanomaterials

The synthesis of transition metal nanoparticles through electron beam irradiation has gained attraction in nanomaterials research due to its rapid processing capabilities and precise control over particle size and morphology. This method facilitates the direct reduction of transition metal cations to metallic nanoparticles without requiring additional reducing agents. In solution-phase synthesis, dilute metal salt solutions are irradiated, leading to the formation of uniform nanoparticles that are particularly suitable for applications in catalysis, electronics, and sensing. Transition metals such as platinum, palladium, and nickel have been effectively synthesized using this approach. Conversely, solid-phase synthesis involves irradiating transition metal precursors in thin films or nanocomposites, resulting in well-dispersed nanoparticles and tailored interactions with the substrate. A major advantage of this technique is the ability to control synthesis parameters, such as electron dose and exposure time, allowing for manipulation of nanoparticle growth and properties. The elimination of chemical residues and the potential for one-step synthesis also enhance its scalability for industrial applications. While the advantages are significant, challenges exist in optimizing the process for practical implementation. Continued research is essential to refine synthesis protocols and explore new transition metal systems. Electron beam irradiation is a promising method for the synthesis of transition metal nanoparticles, offering opportunities for developing advanced nanomaterials for various applications in catalysis, energy, and electronics. Future studies should focus on optimizing these techniques to maximize their industrial applicability. In the following sections, we will briefly describe a few of the most important transition metal and metal compound nanoparticles synthesized by electron beam irradiation.

#### 2.2.1. Gold Nanoparticles

**Figure 3:**
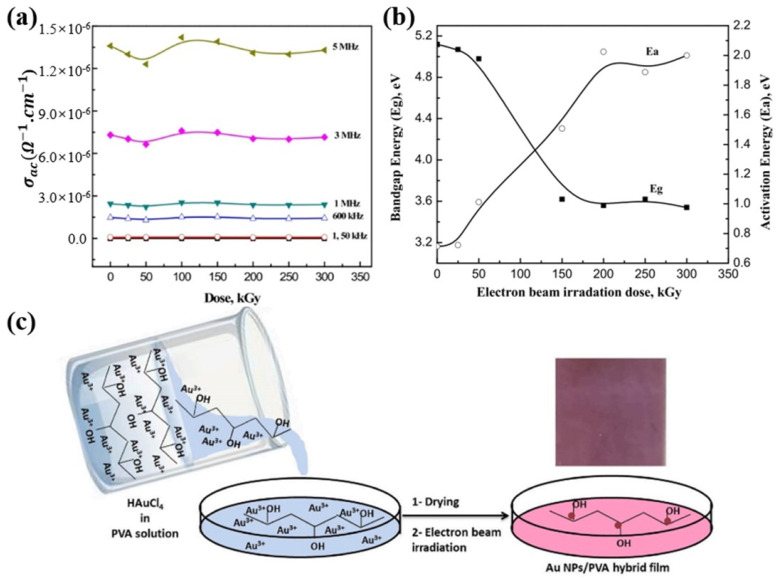
(**a**) Conductivity of specimens, both non-irradiated and irradiated, at ambient temperature. (**b**) Optical band gap and energy need for activation to the electron beam exposure for PVA/Au nanocomposite, before and after irradiation. (**c**) Schematic of the preparation of PVA/Au NPs film, reproduced with permission from ref. [59], Elsevier.

A novel and straightforward strategy has been developed for synthesizing composites of gold nanoparticles (AuNPs) incorporated within polyvinyl alcohol (PVA) films using electron beam irradiation. The PVA/Au nanocomposites exhibited a prominent surface plasmon resonance peak at approximately 540 nm, indicative of the presence of Au nanoparticles. With increasing irradiation doses, the formation of AuNPs was observed, ranging in size from 10 to 65 nm and displaying diverse morphologies. Notably, the optical energy band gap significantly decreased from 5.8 eV for pure PVA to 3.5 eV for samples subjected to a 300 kGy irradiation dose (as shown in Figure 3). Additionally, the incorporation of Au nanoparticles into the PVA matrix, paired with elevated electron beam irradiation, led to a reduction in the thermal stability of the PVA/Au nanocomposite films. AC conductivity measurements at room temperature showed stable behavior, followed by a sharp increase in conductivity as a function of the applied field frequency across various irradiation doses (as shown in Figure 3) [59].

The interest in polymer/metal nanoparticle composites stems from their electrical properties, which closely resemble those of metals, while their processing techniques and mechanical attributes are similar to those of plastics. Consequently, the ability to manipulate the physical and electrical characteristics of these composites is essential for determining their potential applications [60,61]. Additionally, there is an increasing focus on the synthesis of gold nanoparticles (AuNPs) because of their exceptional properties across various research fields. Gold nanoparticles are recognized as the most stable and compatible metal nanoparticles, making them highly suitable for the development of advanced smart devices [62,63]. Kim et al. [64] obtained uniform Au nanoparticles by subjecting gold(I)-alkane thiolate complexes to electron beam irradiation.

On the other hand, poly-metal nanoparticles have also been synthesized by a highly efficient electron beam irradiation process and have good applications in sensor, antimicrobial agents, optical data storage, photo catalysis, and photovoltaics [65,66,67,68]. Y. Ohkubo et al. [69] investigated an efficient method for synthesizing Au-Pd bimetallic nanoparticles with random alloy structures on carbon supports using high-energy electron beam irradiation. This straightforward process involves the reduction of metal precursor ions through radicals formed by radiolysis in an aqueous solution, eliminating the need for surfactants or organic solvents. The researchers discovered that the addition of citric acid and sodium hydroxide, along with careful pH control before irradiation, significantly influences the structural characteristics of the nanoparticles. This was confirmed by advanced analytical techniques such as X-ray diffraction (XRD) and extended X-ray absorption fine structure (EXAFS). By employing metal ion complexing agents and adjusting the solution’s pH, the study highlights a refined approach to achieving tailored bimetallic nanoparticles with desired random alloy structures, showcasing its potential in advancing nanomaterial synthesis. The electron beam radiation condition of the bimetallic nanomaterial synthesis procedure is also reported in the other literature [70,71].

#### 2.2.2. Platinum Nanoparticles

Platinum nanoparticles supported on various oxide substrates, particularly TiO_2_ (Pt/TiO_2_), have garnered significant interest due to their notable chemical properties and effectiveness as catalysts for CO oxidation [72,73]. Satoru Kageyama and colleagues investigated Pt/TiO_2_ composite nanoparticles synthesized via electron beam irradiation [74]. In their study, the synthetic solution was subjected to electron beam irradiation at room temperature for 6.7 s at a dose of 20 kGy and an energy of 4.8 MeV (at the Japan Electron Beam Irradiation Service) to enhance preferential CO oxidation. This innovative method allows for the creation of stabilizer-free Pt/TiO_2_ composite nanoparticles, with the microstructures analyzed using transmission electron microscopy. The resulting Pt nanoparticles, with sizes ranging from 2 to 4 nm, were successfully deposited on TiO_2_ without the addition of stabilizers. The size and morphology of the Pt nanoparticles were significantly influenced by the concentrations of Pt ions and 2-propanol. The catalytic performance for preferential CO oxidation was evaluated across a temperature range of 60 to 140 °C. Remarkably, the Pt/TiO_2_ catalyst featuring spherical Pt nanoparticles achieved a CO conversion rate of 67% with 100% selectivity at a low temperature of 60 °C following electron beam irradiation.

#### 2.2.3. Silver Nanoparticles

**Figure 4:**
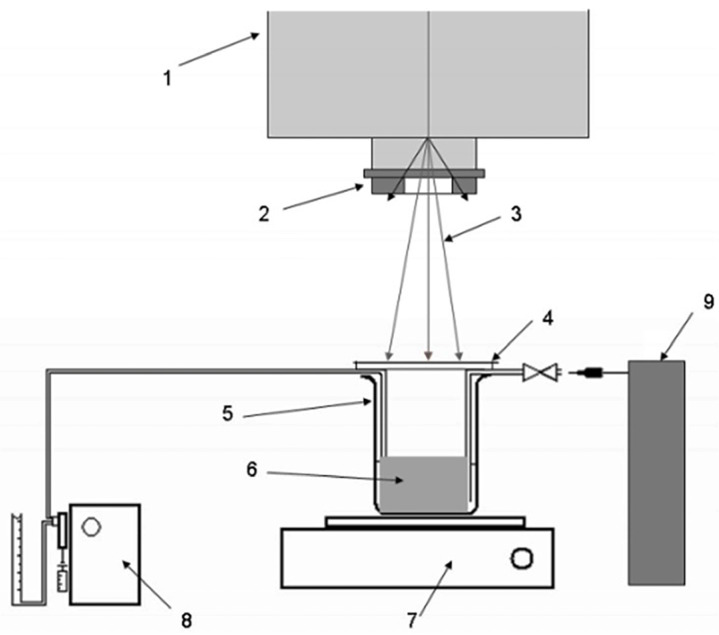
Electron beam (EB) irradiation experimental setup for AgNPs preparation. There are several components, including the following: 1—Exit head for electron accelerators (6 MeV, 2 kGy/min or 5 MeV, 5–10 kGy/s); 2—EB collection monitor in ring shape (for calculating dose rate and accumulating dose); 3—EB field; 4—Al foil; 5—Reaction vessel; 6—Irradiated samples (thickness is adjusted for density and EB energy of the samples); 7—Magnetic stirrer; 8—Pump used to measure precursor solution; 9—Argon bubbling system, reproduced from ref. [75], published by De Gruyter Open Access.

Electron beam (EB) irradiation is an effective approach for producing stable silver nanoparticles (AgNPs) while minimizing the interference from inherent impurities associated with chemical reactions. This prototype experiment utilized linear electron beam accelerators at two different EB absorbed dose rates: 2 kGy/min and 7–8 kGy/s, along with various absorbed dose levels. It established optimal conditions for generating AgNPs through radiolysis alone or through a combination of radiolysis and chemical reduction. A high dose rate is essential for achieving a good yield of AgNPs via radiolysis (as shown in Figure 4), enabling a rapid production process. In contrast, at lower absorbed dose rates, the inclusion of a stabilization agent is recommended. By adjusting the experimental parameters, someone can modulate the balance between chemical and radiolytic reduction processes, allowing for the synthesis of nanoparticles with specific characteristics tailored to various applications [75].

In another investigation, a method combining gamma, electron beam, and synchrotron X-ray irradiation was employed for synthesizing silver nanoparticles in poly (vinyl pyrrolidone) (PVP) [76]. A one-pot synthesis approach was developed to prepare silver nanoparticles in an aqueous PVP solution using synchrotron X-ray radiation. The reduction of metal ions, leading to homogeneous nucleation and nanoparticle formation, is facilitated by hydrated electrons (e_aq_^−^) and hydrogen atom radicals (H^·^), which are generated from the radiolysis of water by synchrotron X-rays. A comparative analysis assessed the effectiveness of this synthesis method against the gamma and electron beam irradiation techniques. The gamma radiation method produced nanoparticles with an average size of 8 nm, while synchrotron X-ray irradiation yielded particles in the range of 10^−15^ nm. Notably, gamma radiolysis and electron beam irradiation resulted in smaller-sized particles with a narrower size distribution compared to those synthesized via X-ray radiolysis. The study also examined the influence of various experimental parameters, such as the concentration of Ag^+^ ions and PVP, on nanoparticle formation.

I. Ion conducted a study on the synthesis of silver nanoparticles embedded in micro-hydrogel particles using electron beam irradiation [77]. The research focuses on creating a hybrid micro-hydrogel that exhibits antibacterial properties. This micro-hydrogel matrix is composed of biocompatible poly(vinyl alcohol) (PVA) that is functionalized with antibacterial agents, specifically graphene oxide and silver nanoparticles. The polymer composites were synthesized through electron beam irradiation at absorbed doses of 10, 25, and 50 kGy to facilitate the formation of silver nanoparticles. The PVA hydrogel itself was produced by irradiating a 20 mL solution of 10% (wt./vol.) PVA with a molecular weight of 400,000 g/mol, in conjunction with graphene oxide and silver salt at a concentration of 1 mg/mL, and additionally functionalized with pyridine and iron salt. F. Yang et al. [78] have developed a method that employs a displacement reaction combined with spontaneous electrolysis to efficiently synthesize switching riddle nanowires of silver tetracyanoquinodimethane (AgTCNQ) over large areas. The authors observed that under electron beam irradiation, silver nanodots and nanoclusters begin to precipitate. The pattern of separation between the Ag nanoclusters and the AgTCNQ matrix resembles that seen in various well-established solid electrolytes, such as Ag_2_S [79], RbAg_4_I_5_ [80], which are commonly used in electrochemical metallization memories or atomic switches [81,82]. In a related study, the authors demonstrated the application of electron beam irradiation for the fabrication of nanoparticles and nanorods on silver surfaces, achieving exceptional nanometric precision. Their work showcased both precise control over the dimensions of the nanorods and high placement accuracy.

**Figure 5:**
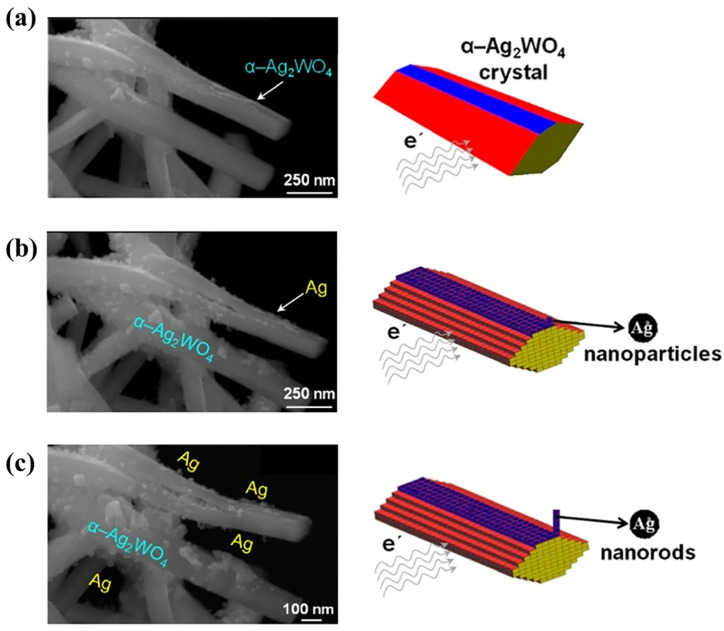
A series of in situ FE-SEM images depicts Ag growth on α-Ag_2_WO_4_ crystals after 30 kV electron exposure without heating. (**a**) The initial image shows the crystal surface before exposure, (**b**) after 6 min, modeled defects indicate the formation of Ag nanoparticles. (**c**) After 10 min, the images reveal the development of Ag nanorods in the same region, reproduced from ref. [86], Nature publication.

The electron beam radiation synthesis of silver nanoparticles was also reported in the other literature [71,83,84,85], which described the electron beam irradiation technique as effective for enabling rapid reactions while eliminating unwanted chemical residues. According to their studies, this method is particularly advantageous because it allows for the controlled growth of silver nanocrystals from dilute solutions of silver nitrate using scanning transmission electron microscopy (STEM) irradiation. The ability to finely tune the initiation and growth processes is crucial for advancing practical applications of this technique, especially in the development of designer nanostructures intended for nano-electronic and nanophotonic applications. Subsequently, achieving precise control over these parameters not only enhances the quality and characteristics of the nanostructures but also accommodates the specific requirements needed for integration into advanced technological platforms. Several research groups have recently made a significant advancement in the production of silver (Ag) nanoparticles and nanowires from various silver-based materials through irradiation with a commonly used electron beam generated by field emission scanning electron microscopy (FE-SEM) or transmission electron microscopy (TEM). This technique involves materials such as α-Ag_2_WO_4_ [86], β-Ag_2_MoO_4_ [87,88], β-AgVO_3_ [89], Ag_3_PO_4_ [90], and Ag_2_CrO_4_ [91]. For the α-Ag_2_WO_4_ case, in situ FE-SEM imaging reveals key insights into the growth of silver (Ag) on α-Ag_2_WO_4_ crystals following 30 kV electron exposure without heating. The initial image captures the crystal surface prior to exposure, establishing a baseline. After 6 min, modeled surface defects emerge, acting as nucleation sites for Ag nanoparticles, indicating that energetic electrons disrupt the crystal lattice. After 10 min, these nanoparticles evolve into Ag nanorods, reflecting the influence of electron interactions on metal morphology (as shown in Figure 5). These findings enhance the understanding of electron-driven growth mechanisms in semiconductor materials, highlighting the interplay between electron exposure and material response, which is crucial for applications in nanotechnology and materials engineering.

**Figure 6:**
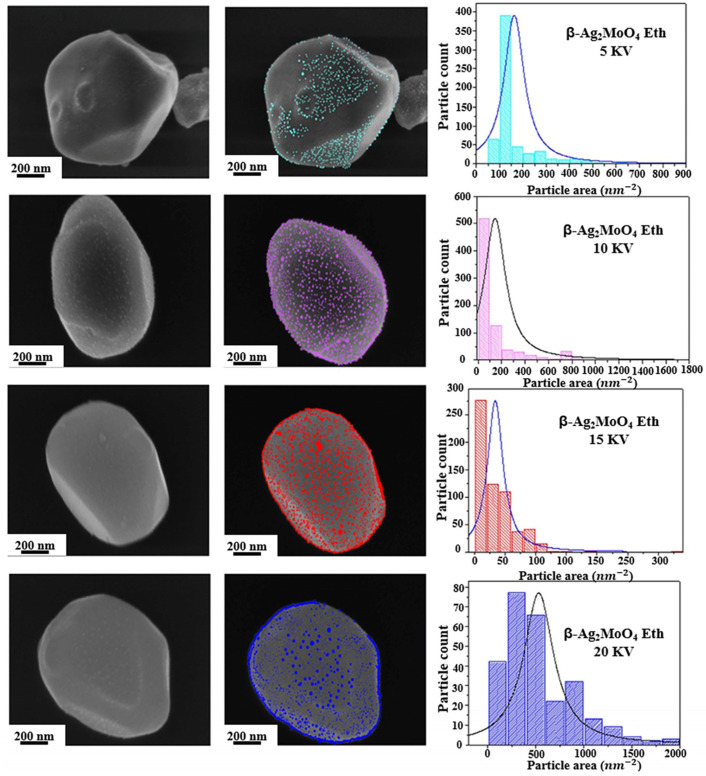
FE-SEM images of β-Ag_2_MoO_4_ microcrystals (**left**) and silver nanoparticle formation on β-Ag_2_MoO_4_ facets at 5, 10, 15, and 20 kV (**right**); with area distribution histograms for silver particles across different voltages after 5 min, reproduced from ref. [88], Taylor & Francis.

Overall, the interaction of these semiconductors with the e-beam facilitates the reduction of Ag cations, leading to the formation and growth of metallic Ag on their surfaces, which results in Ag nanoparticles associated with semiconductors (as shown in Figure 6). Several recent publications have discussed the mechanisms underlying the formation of these new structures [92,93]. For instance, Lin et al. [94] investigated the electronic reconstruction of α-Ag_2_WO_4_ nanorods and their potential applications in visible-light photocatalysis. Meanwhile, studies by Xu et al. [95] and Thomas et al. [96] focused on evaluating the photocatalytic activity of α-Ag_2_WO_4_ particles and nanoparticles, respectively. Additionally, Sreedevi et al. [97] analyzed the impact of 8 MeV e-beam irradiation on the structural and optical properties of α-Ag_2_WO_4_ nanoparticles, emphasizing the concurrent growth of Ag nanoparticles surfaces.

**Figure 7:**
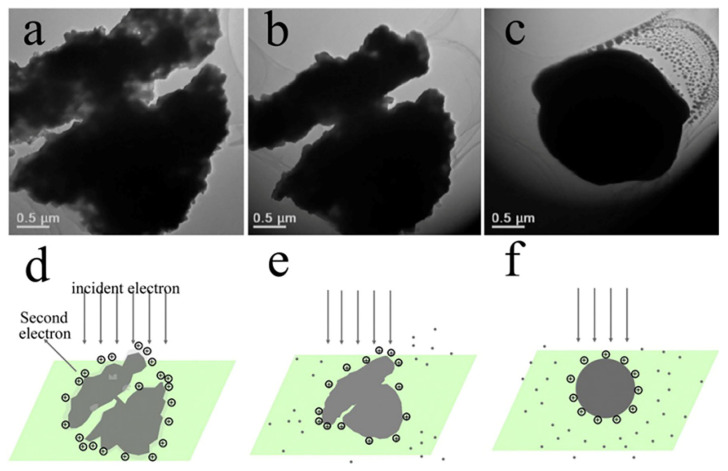
(**a**–**c**) TEM images showing different stages of a precursor under different EBCD. (**d**–**f**) Schematic diagrams showing Ag atoms escaping from the precursor, reproduced with permission from ref. [101], Elsevier.

Silver nanoparticles are extensively utilized as optical labels due to their sensitivity to surface-enhanced Raman scattering effects [98,99,100]. J. Gong conducted an in situ synthesis of Ag nanoparticles using electron beam irradiation within the transmission electron microscope (TEM) chamber at room temperature, thoroughly exploring the growth mechanism [101]. The size of the Ag nanoparticles was precisely controlled by adjusting the electron beam current density. Two distinct growth stages were identified: the initial stage was dominated by the discharging effect, while the subsequent stage was influenced by the heating effect. This nanoparticle synthesis technique may be applicable to the fabrication of other metallic nanoparticles as well. In the synthesis of Ag nanoparticles, the precursor initially appeared fluffy with an electron beam current density (EBCD) of 5 × 10^3^ A/m^2^ (as shown in Figure 7a). Increasing the EBCD to 1.5 × 10^4^ A/m^2^ caused significant shrinking of the precursor (as shown in Figure 7b), but no further alterations occurred at this level over an extended duration, similar to the behavior recorded during nanoparticle growth. As the EBCD increased to 6 × 10 A/m^2^, the precursor transformed into a compact sphere (as shown in Figure 7c). A proposed growth mechanism, illustrated in Figure 7d–f, implies that when the electron beam irradiates the precursor, electron–nucleus interactions ionize some atoms, resulting in a positively charged electrostatic field. Notably, this synthesis process does not require surfactants, making it a streamlined approach for producing Ag nanoparticles.

### 2.3. Other Metal Nanomaterials

**Figure 8:**
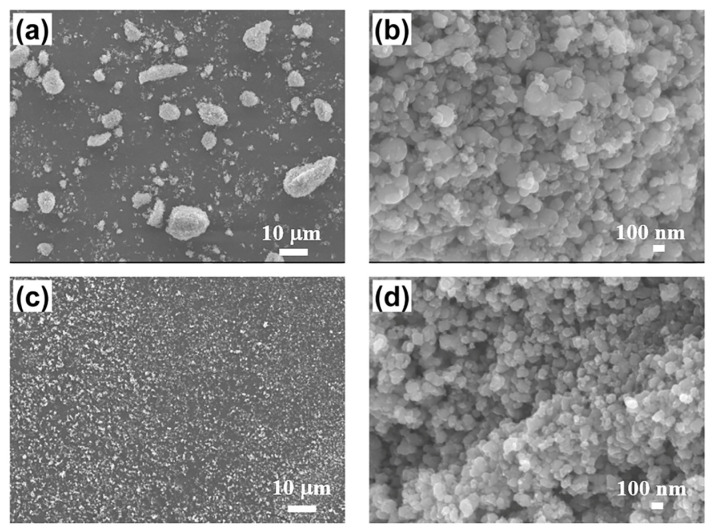
SEM images of (**a**,**b**) the hydrolysis synthesized TiO_2_ nanoparticles and (**c**,**d**) the E-beam irradiated TiO_2_ nanoparticles, reproduced from ref. [113], The Korean Electrochemical Society.

Apart from nanoparticles, electron beams in TEM have been adeptly utilized to generate and control a variety of nanostructures. For instance, nanoscale gallium (Ga) particles were produced by triggering a charging explosion via in situ electron beam irradiation on silica-coated Ga microspheres [102]. Similarly, the conversion of indium sulfide (In_2_S_3_) nanosheets into indium oxide (In_2_O_3_) nanocrystals was achieved through electron beam exposure, inducing chemical bond alterations within In_2_S_3_ [103]. Moreover, sodium nanostructures were noted because of irradiating NaCl powders [104]. Wang et al. [105] reported the synthesis of single-crystalline Cu nanorods via electron beam irradiation, outlining a mechanism that manipulates the electron exposure on Cu powders situated on carbon films. Yen et al. [106] investigated how Cu nanoparticles evolved from CuCl through in situ TEM irradiation. Copper-containing materials, such as zeolites, can also produce nanorods with high aspect ratios [107]. Furthermore, Padhi et al. [108] demonstrated that electron beam irradiation can transform Cu_2_(OH)_3_NO_3_ nanoflakes into nanocrystalline CuO. Various growth processes driven by electron beams highlight their potential for technological applications, including those involving cobalt and indium [109,110]. Del Angel et al. [111] introduced the in situ creation of nickel nanoparticles by targeting NiO/ZrO_2_-CeO_2_ and NiO with an electron beam, while Huang et al. [112] examined the impacts of irradiation on Ti_3_AlC_2_ samples, noting that aluminum was primarily sputtered without inducing amorphization. Ahn et al. [113] have developed a novel synthesis method for TiO_2_ nanoparticles using electron beam (E-beam) irradiation, specifically aimed at enhancing their application as anode materials in lithium-ion batteries. This method successfully produces small TiO_2_ nanoparticles with a narrow particle size distribution (as shown in Figure 8). The study investigates the effects of E-beam irradiation on both the synthesis of these nanoparticles and their electrochemical performance as alternative anode materials for Li-ion batteries. The findings reveal that TiO_2_ nanoparticles generated via E-beam irradiation exhibit superior cycling performance and rate capability compared to those synthesized through conventional hydrolysis methods. This enhanced electrochemical performance is primarily attributed to the smaller particle size and narrow size distribution, which together increase the surface area, providing a greater number of reaction sites and reducing the diffusion length for Li^+^ ions within the TiO_2_ nanoparticles. This research highlights the potential of E-beam irradiation as an effective approach for producing high-performance TiO_2_ anode materials in lithium-ion batteries. Gonzalez-Martinez et al. [114] recently proposed a catalyst-free method for the room-temperature growth of crystalline aluminum borate nanowires using e-beam irradiation in a transmission electron microscope (TEM). An increasing number of studies have observed the formation of lead (Pb) nanoparticles through e-beam irradiation on various lead halide perovskites, including CH_3_NH_3_PbX_3_ [115,116], (C_4_H_9_NH_3_)_2_PbBr_4_ [117], and CsPbX_3_ [118,119].

In a notable study, Kim et al. [120] synthesized Bi nanoparticles by irradiating BiCl_3_ films using TEM under extreme conditions at a high voltage of 400 kV. Conversely, Sepulveda-Guzman et al. [121] demonstrated the formation of Bi nanoparticles from NaBiO_3_ precursors in a TEM without prior treatment and under milder conditions. They hypothesized that the radiolysis process decomposes NaBiO_3_, yielding Bi^5+^ ions and neutral Bi^0^ that cluster into small crystalline seeds, which then grow into larger nanoparticles through Ostwald ripening. Recently, Chang et al. [122] reported in situ observation of Bi nanoparticles growing on BiOCl photocatalysts using real-time TEM. Their findings highlighted how e-beam irradiation accelerates nanoparticle growth while examining the associated crystallinity and elemental changes occurring during the synthesis.

**Figure 9:**
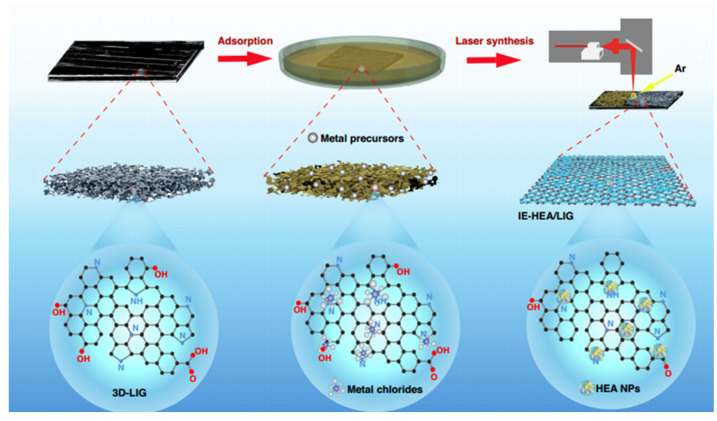
Schematic diagram of laser synthesis for CrMnFeCoNi high-entropy alloy (HEA) nanoparticles and iron-doped HEA/lignin composite (IE-HEA/LIG), reproduced from ref. [123], Nature Publication.

A multi-metal CrMnFeCoNi nanoparticle synthesis was reported by Y. Liu et al. [123]. They showed the effective laser solid-phase synthesis of CrMnFeCoNi nanoparticles by irradiating mixed metal precursors on a laser-induced graphene (LIG) support featuring a three-dimensional (3D) porous structure. The resulting CrMnFeCoNi nanoparticles are encapsulated within multiple layers of graphene, forming graphene shell-encapsulated high-entropy alloy (HEA) nanoparticles. This study investigates the mechanisms involved in the laser solid-phase synthesis of HEA nanoparticles on LIG supports through a combination of theoretical simulation and experimental observations. Key processes considered include the adsorption of mixed metal precursors, thermal decomposition, reduction via electrons from laser-induced thermionic emission, and the splitting of liquid beads. Remarkably, the production rate achieved with the current laser setup is as high as 30 g/h. The laser-synthesized graphene shell-encapsulated CrMnFeCoNi nanoparticles (as shown in Figure 9), when loaded on LIG-coated carbon paper, are utilized as binder-free integrated 3D electrodes. These electrodes demonstrate excellent electrocatalytic activity for the oxygen evolution reaction, achieving an overpotential of 293 mV at a current density of 10 mA/cm^2^, along with exceptional stability over 428 h in alkaline media. This performance surpasses that of commercial RuO_2_ catalysts and other relevant catalysts synthesized using alternative methods. Additionally, this work highlights the versatility of the laser synthesis technique, successfully producing CrMnFeCoNi oxide, sulfide, and phosphide nanoparticles.

### 2.4. Carbon-Based Nanocomposite

Boostani and colleagues examined the use of powder metallurgy to create SiC nanoparticles and graphene nanosheets, which were utilized as thermally active materials in aerospace applications. These metal matrix nanocomposites showed exceptional mechanical qualities, such as enhanced tensile elongation, particularly in aluminum matrices reinforced with ceramic nanoparticles, and outperforming conventional strengthening methods. The use of nano ZrO_2_ particles also helped decrease deterioration caused by atomic oxygen (AO) in metal nanocomposites [124,125,126]. Uddin and others focused on developing flame-retardant nanocomposite coatings for aircraft applications [127]. They synthesized graphene oxide (GO) using the Hummers method and applied it through a layup technique followed by vacuum bagging. Burn tests indicated that the burn length was shorter when nanocomposites were included. Although these approaches target the aerospace industry, the application of fused deposition modeling (FDM) is limited by its decreased material strength. Carbon-based materials such as carbon nanotubes and graphene also benefit from this technique, as e-beam irradiation can modify their properties to improve electrical and thermal conductivities. However, challenges such as equipment costs and scalability need addressing for broader adoption. Despite these hurdles, the precision and versatility offered by e-beam irradiation make it a valuable tool in advancing nanomaterial research and technology, underpinning its potential to revolutionize the field of nanotechnology. As research progresses, further refinement of this technique is expected to enhance its application scope, driving innovation across diverse scientific and industrial domains.

## 3. Synthesis of Nanomaterials by Gamma Radiation

**Scheme 5:**
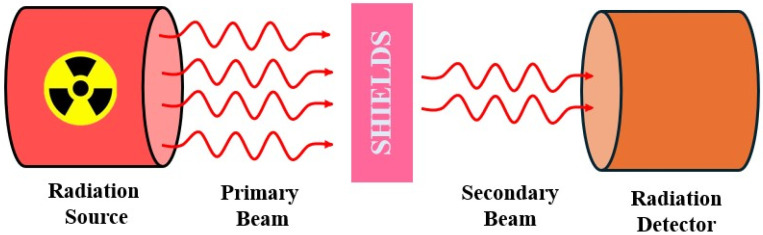
Experimental setup of gamma radiation synthesis.

Gamma radiation stands as a potent and innovative methodology in the synthesis of nanomaterials, significantly contributing to advancements in the field of nanotechnology. This technique leverages the unique properties of gamma rays particularly their high energy, deep penetration, and uniform energy distribution to induce chemical reactions that lead to the formation of nanoparticles [128]. One of the primary benefits of using gamma radiation is its ability to initiate these reactions without the necessity of adding chemical catalysts or reducing agents. This absence of additional chemicals not only reduces the risk of contamination but also aligns with the principles of green chemistry, making the process more environmentally friendly compared to conventional synthesis methods [129]. The experimental setup of gamma radiation synthesis is shown in Scheme 5.

Gamma ray irradiation has been shown to exhibit a bonding effect that plays a significant role in the synthesis and properties of various materials. This effect refers to the alterations in chemical bonds and interactions that occur when materials are exposed to gamma radiation. During gamma ray exposure, the energy from the radiation can cause ionization and excitation of atoms and molecules, leading to the breaking and forming of chemical bonds. This process can facilitate the reduction of metal ions, enhance cross-linking in polymers, and promote the formation of nanoparticles or composites with improved structural integrity. In the context of nanomaterials synthesis, gamma ray irradiation can lead to the controlled formation of size and shape, as well as the distribution of nanoparticles, by influencing the bonding interactions between the nanoparticles and the supporting matrix or substrate. Additionally, the resulting materials often exhibit enhanced physical and chemical properties, such as increased stability, conductivity, and reactivity, making them suitable for applications in fields like catalysis, energy storage, and environmental remediation.

**Scheme 6:**
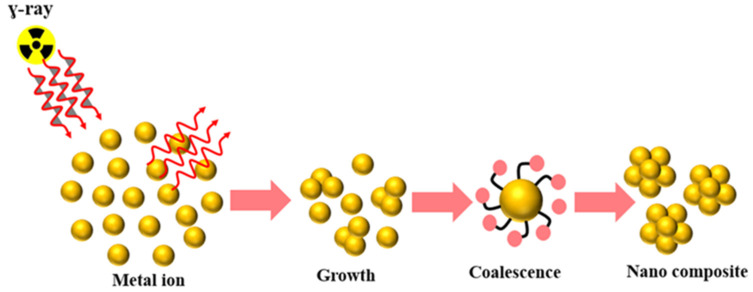
Mechanism of metal nanoparticle synthesis by gamma ray irradiation.

**Scheme 7:**
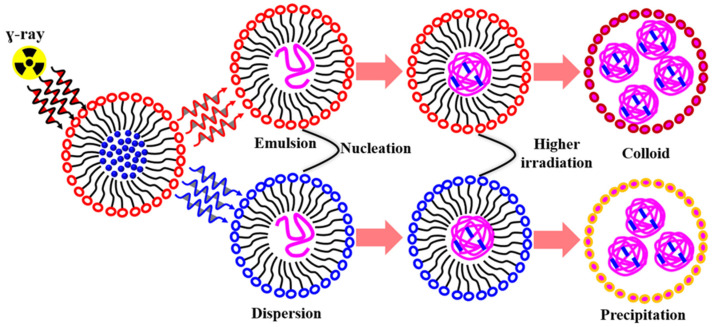
Gamma ray-induced synthesis process of polymeric nanoparticles.

In the synthesis of metallic nanoparticles, gamma radiation is particularly effective. For example, the radiolytic reduction of metal ions such as silver (Ag^+^) and gold (Au^3+^) can result in the formation of finely dispersed metal nanoparticles, which are integral in various industrial and biomedical applications [130]. Silver nanoparticles produced in this manner are renowned for their antimicrobial properties and are used extensively in medical devices, textiles, and coatings to inhibit microbial growth. Similarly, gold nanoparticles synthesized through gamma radiation hold significant potential in fields ranging from drug delivery to cancer treatment, as well as in the development of biosensors due to their excellent conductivity and biocompatibility. Beyond metals, gamma radiation plays a crucial role in the modification and enhancement of polymer materials. By inducing cross-linking in polymers, it can result in the formation of polymer nanocomposites, which exhibit superior mechanical strength, thermal stability, and chemical resistance [131]. These enhanced properties expand the application range of polymers, making them suitable for use in areas such as aerospace, automotive, and electronics. The ability to control the extent of cross-linking with precision further allows for the tailoring of material properties to meet specific requirements. Moreover, gamma radiation can modify existing nanostructures, altering surface properties or creating defects strategically, thus making them more suitable for targeted applications [132]. For instance, introducing defects can enhance the catalytic activity of certain nanomaterials, broadening their use in chemical reactions or environmental remediation. This versatility and adaptability of the gamma radiation technique underscores its importance in the nanotechnology landscape, where precise control over material properties is essential. Gamma radiation-induced synthesis represents a versatile and sustainable approach in the development of nanomaterials, fostering advancements across multiple disciplines. The method’s capacity to produce a wide range of nanoparticles with controlled properties makes it a critical tool in the continuous evolution of nanotechnology, promoting innovations that address both current and future scientific and industrial challenges [133]. As the demand for efficient and environmentally conscious synthesis methods grows, the role of gamma radiation in nanomaterial synthesis is likely to expand further, entrenching its value in the pursuit of technological breakthroughs. The mechanisms of metal nanoparticles’ and polymeric nanoparticles’ formation by gamma radiation are shown in Scheme 6 and Scheme 7, respectively.

### 3.1. Metal Nanoparticles and Nanocomposites

Nanostructures have gained significant attention in the biology research area for their antimicrobial applications due to their unique properties and enhanced reactivity compared to bulk materials. Various forms of nanostructures, such as metallic nanoparticles (e.g., silver, gold, copper), carbon nanotubes, and nanocomposites, exhibit potent antimicrobial effects against a wide range of microorganisms. These nanostructures operate through several mechanisms, including the generation of reactive oxygen species (ROS), disruption of microbial cell membranes, inhibition of biofilm formation, and the release of toxic metal ions. Their ability to efficiently target and eliminate pathogens makes them suitable for applications in medical devices, coatings, wound dressings, and filtration systems, ultimately contributing to improved infection control and environmental sanitation [134,135,136].

**Figure 10:**
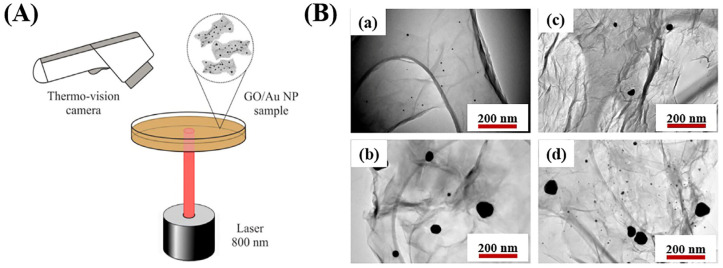
(**A**) Experimental setup for the determination of photothermal properties of GO/Au NP composites. (**B**) TEM images of GO/Au NP composites prepared at (**a**) 1 kGy, (**b**) 5 kGy, (**c**) 10 kGy, and (**d**) 20 kGy irradiation dose, reproduced with permission from ref. [139], Elsevier.

Gamma irradiation techniques have enabled the successful fabrication of various nanometals and their corresponding polymer composites. Notably, colloidal silver and gold nanoparticles (NPs) have been synthesized from metal salts in aqueous solutions using a ^60^Co gamma-ray source. When assessing the efficacy of this method compared to traditional chemical reduction, silver NPs produced via gamma irradiation demonstrated significantly higher concentrations and a narrower size distribution. For gold NPs, however, both methods yielded comparable results. Specifically, the gamma irradiation of a 1.0 × 10^−3^ M AgNO_3_ solution resulted in silver colloids that were nearly 100 times more concentrated than those achieved through citrate reduction. Furthermore, increasing the AgNO_3_ concentration to 2.0 × 10^−2^ M further enhanced silver colloid concentrations via the radiation method [137,138]. Alternative precursors, including silver sulfate and silver chloride, have also been employed for synthesis. Additionally, a distinctive nanostructural hybrid particle powder composed of Ag-TiO_2_ was created through a combination of gamma irradiation and hydrothermal treatment. This innovative approach enhanced visible luminescence, which is attributed to the inter-band transitions within TiO_2_ nanoparticles, facilitated by energy transfer from the silver nanoparticles [138]. The synthesis of graphene oxide/gold nanoparticle (GO/Au NP) composites via gamma-ray irradiation has shown promising results for nanomaterial production. This method is an efficient alternative to conventional gold nanoparticle synthesis (as shown in Figure 10A), being simple, rapid, and cost-effective. The low doses of gamma irradiation (1–20 kGy) were employed to anchor gold nanoparticles onto graphene oxide sheets by D.P. Kepi’c et al. [139]. GO was selected because of its large surface area and excellent dispersibility due to oxygen-containing functional groups. Gamma irradiation effectively reduced chloroauric acid, resulting in uniformly distributed spherical Au NPs on the GO surface while simultaneously reducing GO and partially restoring its structure. The Au NPs synthesized at 1 kGy exhibited sizes up to 20 nm, whereas higher doses (5 and 10 kGy) led to larger particles, and the highest dose of 20 kGy resulted in a broad size range from several nanometers to 120 nm (as shown in Figure 10B). Ni^2+^ ions in an aqueous solution attempt to synthesize freestanding nickel nanoparticles by gamma radiation. During the process, a black precipitate formed, which we identified as metallic nickel particles due to their observable attraction to a strong magnet. This magnetic response suggested the presence of nickel, as the particles took on unique shapes within the solution. Further investigation using transmission electron microscopy (TEM) revealed that the size of the resulting particles was approximately 2 nm [140]. Recent research explored the synthesis of freestanding nickel nanoparticles using gamma irradiation from a MDS Nordion 1000 Elite Cs-137 source at a dose rate of 0.12 Gy s^−1^ [141]. By reducing nickel chloride (NiCl_2_) in an aqueous solution, metallic nickel nanoparticles were produced. These nanoparticles exhibited ferromagnetic-like behavior, with magnetization approximately 30% lower than that of bulk nickel. Additionally, they showed a high electrochemically active surface area (ECSA) of 39.2 m^2^ g^−1^ and good electrochemical reversibility, indicating their potential for energy storage applications. Overall, synthesized nickel nanoparticles are promising candidates for energy storage devices, thanks to their unique magnetic properties and effective electrochemical characteristics, warranting further investigation into their practical applications.

**Figure 11:**
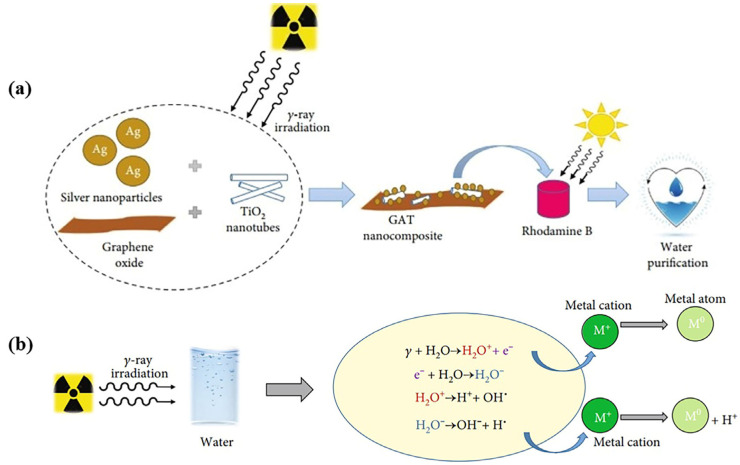
(**a**) Concept developing the GAT nanocomposite to facilitate the photodecomposition of rhodamine B. (**b**) Gamma irradiation-induced water radiolysis and the subsequent formation of metal atoms, reproduced from ref. [142], Wiley & Sons.

Recent studies have investigated the gamma irradiation-assisted synthesis of silver nanoparticle-embedded graphene oxide-TiO_2_ nanotube (GAT) nanocomposites for the photodegradation of organic dyes. The research demonstrated that gamma irradiation significantly enhances the composite synthesis process. The resulting GAT nanocomposites exhibited impressive efficiency in degrading Rhodamine B (RhB) after just 60 min of exposure to natural sunlight, as confirmed by UV–Vis absorption spectroscopy. These findings underscore the potential of gamma irradiation as a clean and controllable method for fabricating valuable nanocomposite materials aimed at wastewater purification and other environmental applications. The study also explored the influence of the irradiation process, examining both one-step and two-step methodologies at varying doses (5, 10, 15, 20, and 25 kGy) on the physicochemical properties of the GAT nanocomposites. Overall, the results highlight the promise of this approach in developing effective solutions for environmental remediation [142]. Figure 11a,b depict the GAT nanocomposite employed for the photodegradation of rhodamine B and the generation of metal atoms resulting from water radiolysis induced by gamma irradiation.

**Figure 12:**
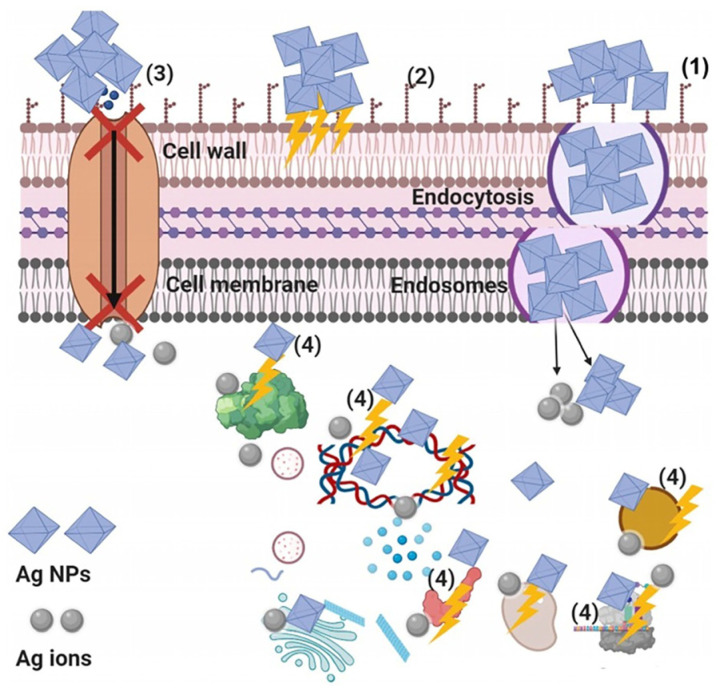
Schematic overview of the antibacterial action of PVP@D-AgNs: (1) They adhere to bacterial surfaces, disrupting membranes. (2) They generate reactive oxygen species (ROS), weakening the cell wall. (3) They block ion movement. (4) They enter cells, impair organelles, and cause cell lysis, reproduced from ref. [143], Spinger.

In the study conducted by M. Bekhit et al., a decahedron-like silver nanostructure (D-AgNs) was successfully synthesized in an aqueous solution using gamma-radiolysis, with polyvinylpyrrolidone (PVP) serving as a capping agent, eliminating the need for a separate reducing agent [143]. The UV–Vis absorption spectra displayed prominent surface plasmon resonance (SPR) bands between 350 and 600 nm, confirming the successful formation of colloidal D-AgNs. Furthermore, the data indicated that these synthesized D-AgNs exhibited remarkable antimicrobial and antibiofilm properties, making them suitable for applications across different fields. Notably, they show promises for disinfecting wastewater contaminated with harmful pathogenic microbes, as well as for biomedical applications, particularly in combating bacteria responsible for urinary tract infections (UTIs) and unicellular fungi (as shown in Figure 12).

Mohamed Bakr Mohamed and M.H. Abdel-Kader [144] reported that the crystallite size of ZnS increased from 4 nm to 10 nm as the annealing temperature rose from 300 to 500 °C. For the PVA/ZnS nanocomposite, the addition of ZnS nanoparticles improved the extinction coefficient at 300 °C, but this enhancement diminished with further increases in annealing temperature. According to S. Sugumaran et al. [145] study, the optical transmittance of PVA/Al_2_O_3_ thin films was approximately 80% for the as-grown films, which improved with higher annealing temperatures. The band gap energy decreased from 3.74 to 3.78 eV with increasing temperature, and the dielectric constant values ranged between 8 and 16, exceeding those of pure PVA. The dielectric loss values were determined to be between 0.1 and 0.6.

### 3.2. Metal Perovskites Nanocomposites

**Figure 13:**
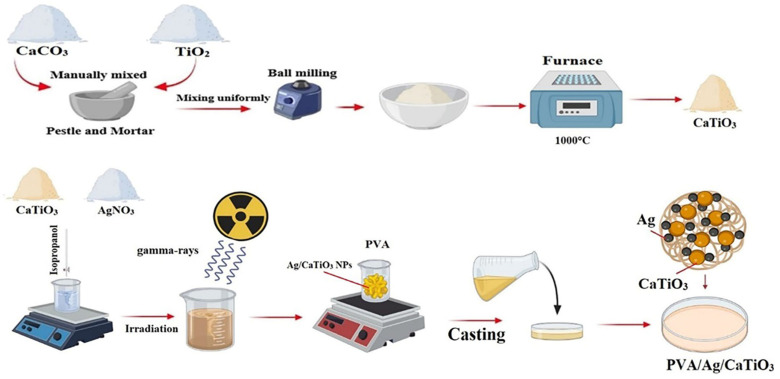
Schematic representation of the PVA/Ag/CaTiO_3_ nanocomposite film synthesis, reproduced from ref. [147], Nature publication.

Numerous prior studies have emphasized the characteristics and potential applications of various perovskites. Materials such as SrZrO_3_, SrRuO_3_, CaGeO_3_, PbTiO_3_, SrTiO_3_, BaTiO_3_, GdFeO_3_, and CaTiO_3_ have garnered attention, especially calcium titanate (CaTiO_3_), due to its exceptional optoelectronic, ferroelectric, and photocatalytic properties [146]. CaTiO_3_ is categorized as an n-type semiconductor and possesses a perovskite structure. A nanocomposite film was developed from polyvinyl alcohol (PVA), silver nanoparticles, and calcium titanate (CaTiO_3_) using gamma radiation-induced reduction methods. The study explored how the film’s structural, optical, direct current (DC) electrical conductivity, and dielectric properties varied with temperature (as shown in Figure 13). It was observed that as the temperature increased, the average crystallite sizes of CaTiO_3_ and silver nanoparticles decreased from 19.8 to 9.7 nm and from 25 to 14.8 nm, respectively, while the optical band gap rose from 5.75 to 5.84 eV at 373 K [147]. Another study showed the application of synthesized novel PVA/Ag/CaTiO_3_. Gamma radiation is utilized for synthesizing nanomaterials in optoelectronics, allowing for precise control over material properties and uniform nanostructure formation while reducing hazardous chemicals. This method enhances the purity of quantum dots for displays, thin films for LEDs, and up conversion materials, offering significant potential to improve the performance of optoelectronic devices [148,149].

### 3.3. Carbon-Based Nanomaterials

Graphene-based nanocomposites combine graphene with other materials to enhance properties such as mechanical strength, electrical conductivity, and thermal performance. These composites leverage graphene’s high surface area and unique characteristics, making them suitable for applications in electronics, energy storage, automotive, and biomedical fields. Manufacturing methods include solution mixing and in situ polymerization, but challenges remain in achieving uniform dispersion and scalability. The ongoing research aims to maximize the benefits of these advanced materials for various practical uses [150,151,152]. A recent study investigates the shielding capabilities of a lightweight graphene-based composite with a density of approximately 1 g/cm^3^. The findings indicate that the linear attenuation coefficient is dependent on radiation energy, corroborating the predictions made by the XCOM model. Additionally, the mass attenuation coefficient for selected radiation energies is comparable to that of established shielding materials, exceeding 0.2 cm^2^/g at higher energy levels. This research supports the effectiveness of the XCOM model in predicting gamma and X-ray radiation attenuation for novel materials, positioning the graphene-based composite as a viable candidate for radiation shielding applications [153].

## 4. Ionizing Radiation Advantages

Ion radiation, such as gamma and electron irradiation, plays a crucial role in defect engineering within 2D materials, offering a powerful method to modify and enhance their properties for various applications. Gamma rays, with their high-energy photons, can penetrate deeply into materials, inducing lattice defects by displacing atoms and generating vacancies or interstitials. These defects can significantly alter electronic, optical, and chemical properties, making gamma irradiation a versatile tool for material modification. Similarly, electron irradiation allows for precise control over defect creation due to its capability to focus the irradiation at targeted areas, thereby introducing specific types of defects. By adjusting parameters such as radiation dose and energy, researchers can tailor the number and type of defects, enabling customized tuning of characteristics such as electrical conductivity, catalytic activity, and mechanical strength in 2D materials. This ability to engineer material properties at the atomic scale opens new avenues for developing advanced materials in fields including electronics, optoelectronics, and quantum computing. A deep understanding and precise manipulation of these defects are essential for maximizing the functionality of 2D materials. Hence, recent research has focused extensively on observing, understanding, and controlling defects in 2D materials. The most basic defect in any material is a single vacancy, where a lattice site is vacant. In transmission electron microscopy (TEM), single electron impacts can be treated as separate events since typical electron fluxes ensure that just one electron is present in the microscope column at any given time. The low mass of electrons limits kinetic energy transfer to a few electron volts, so in a pristine crystal, such knock-on damage usually results in single vacancies. In 2D materials like graphene, vacancy-type defects are the prevalent structural changes observed under electron irradiation, while transformations involving bond rotation are uncommon, though they can occur in some instances [154]. In hexagonal boron nitride (h-BN), electron irradiation forms nitrogen-terminated triangular pores [155,156]. J. Kotakoski and colleagues investigated point defects in transitioning from graphene to two-dimensional amorphous carbon using electron beam irradiation [157]. Electron damage to materials is notably significant due to the dominant electron-based characterization methods. Elastic and inelastic scattering events lead to six primary damage mechanisms: displacement, sputtering, heating, charging, radiolysis, and radiation-induced contamination [158]. In metals, damage principally arises from sputtering and displacement, whereas in insulators and semiconductors, it mainly results from radiolysis and charging [159,160].

Common advantages of ionizing radiation for nanomaterials synthesis, such as the following:

- Controlled modification in ionic radiation allows for precise manipulation of materials at the nanoscale, facilitating the controlled growth and structuring of nanomaterials.
- Chemical transformation for the high energy of ion beams induces significant chemical changes, leading to enhanced material purity and the formation of desired nanostructures.
- Enhanced properties of ionic radiation can improve the electrical, thermal, and mechanical properties of nanomaterials, making them more suitable for a variety of applications.
- Surface characterization of his technique enables fine-tuning of surface properties, which is crucial for applications in electronics, catalysis, and nanomedicine.
- Innovation in nanotechnology by expanding the toolbox for nanomaterial synthesis, ionic radiation represents a critical advancement that drives research and innovation in the field.

**Figure 14:**
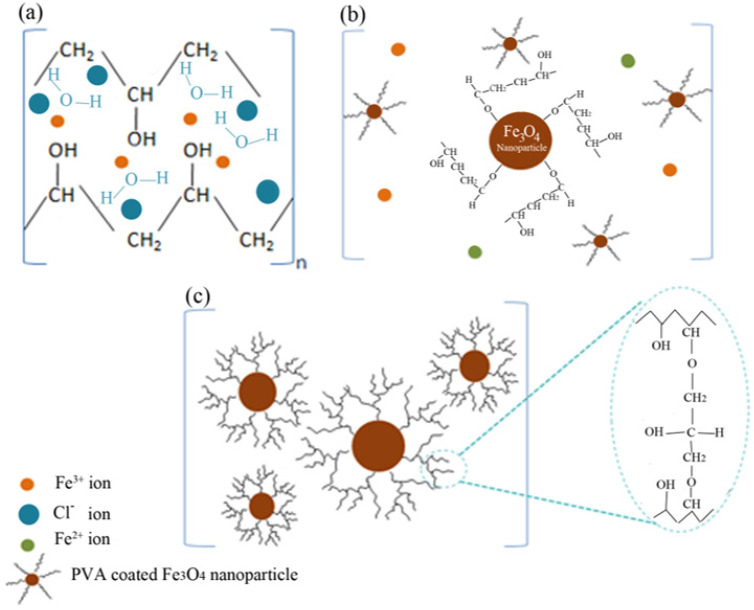
The proposed mechanism for the formation of PVA- Fe_3_O_4_ nanoparticles: (**a**) PVA interactions with Fe^3+^ ions before irradiation; (**b**) PVA interactions with Fe_3_O_4_ surfaces post-irradiation; (**c**) coating of Fe_3_O_4_ nanoparticles via polymer crosslinking at high radiation doses, reproduced from ref. [162].

The synthesis of metallic nanoparticles via gamma irradiation offers notable advantages over traditional methods, including the production of highly pure nanoparticles without by-products or chemical-reducing agents, and precise control over size and structure found that isopropanol acts as a scavenger for OH^•^ and H^•^ radicals while forming secondary radicals that reduce metal ions (M^+^) to their zero-valent state (M^0^) during irradiation. Additionally, multivalent metal ions undergo reduction through a multi-step process, potentially involving disproportionation of lower valence states due to irradiation [71,161]. Colloidal metal oxide nanoparticles are tiny particles made from metal oxides, typically measuring between 1 and 100 nanometers. These nanoparticles are dispersed in a liquid medium, resulting in a colloidal solution. Commonly used metal oxides include titanium dioxide, zinc oxide, iron oxide and copper oxide, known for their distinct optical, electrical, magnetic, and catalytic properties at the nanoscale. Due to their increased surface area and reactivity compared to bulk materials, these nanoparticles are highly valuable across numerous applications such as catalysis, environmental cleanup, sensing, drug delivery, and photocatalytic processes. Their characteristics can be precisely adjusted through modifications in size, shape, and surface properties. As a result, they play a crucial role in advancing research in nanotechnology, materials science, and biomedical fields. A notable synthesis method for these nanomaterials is the gamma-radiolysis technique. Alam Abedini et al. [162] successfully produced colloidal Fe_3_O_4_ nanoparticles using this method in an aqueous solution of iron chloride, employing polyvinyl alcohol as a stabilizer and isopropanol as a hydroxyl radical scavenger, as shown in Figure 14.

The synthesis of nanomaterials through electron beam and gamma ray irradiation depends on various factors such as dose rate, contact time, and the source of radiation [163,164,165]. The detailed impact of different conditions for gamma and electron beam irradiation in the synthesis of nanomaterials is illustrated in Table 1.

## 5. Major Fields of Nanomaterial Application

### 5.1. Health and Medical Care

In magnetic resonance imaging (MRI), the use of metal nanoparticles has been found to be useful as contrast agents, which can aid in the visualization of different types of tissues. For example, detecting cancer in the lymph nodes and liver can be accomplished using superparamagnetic iron oxide nanoparticles [1]. Computed tomography (CT) scans can benefit from gold nanoparticles, which can improve contrast and be used to locate tumors more efficiently. Additionally, gold nanoparticles can be used in fluorescence imaging for real-time monitoring of cancer cells [166]. Furthermore, gold NPs can be used in wound dressings and coatings to prevent bacterial infections [167]. Iron oxide nanoparticles and Ag nanoparticles are potent agents against monkey pox, functioning by chelating circulating viruses in the bloodstream and subsequently blocking virus host cell binding and penetration [168]. There is growing evidence that silver nanoparticles possess strong antimicrobial properties which make them ideal for the treatment of infections caused by viruses, bacteria, and other microorganisms [169].

### 5.2. Drug Delivery

Studies suggest that nanoparticles can cross the blood–brain barrier with the aid of hyperosmotic agents like Mannitol, enhancing their therapeutic effectiveness [170]. Active targeting involves linking specific molecules to drug delivery systems to accurately deliver drugs to tumors, within cancer cells, or specific molecules [171]. Gold nanoparticles (AuNPs) are particularly notable in drug delivery due to their small size, large surface area, and ease of modification, which provide precise targeting, fewer side effects, and higher drug efficacy. The stability and biocompatibility of gold nanoparticles further enhance their use in both therapeutic and diagnostic applications, demonstrating considerable advancements in targeted medicine having unique absorption properties in photothermal therapy to selectively destroy cancer cells while preserving healthy tissue [172]. Mesoporous silica nanoparticles have proven to be innovative drug delivery systems that can also combat bacterial resistance, exhibiting versatility and potential in various medical treatments [173]. Lohiya, G. et al. developed doxorubicin-loaded, chitosan-coated, aptamer-mesoporous silicon nanoparticle bioconjugates specifically to target breast cancer cells with overexpressed EGF receptors [174]. Carbon-based materials are extensively used in technological applications, especially for drug delivery, due to their excellent drug-loading capabilities. These nanomaterials offer high biostability, biocompatibility, and reduced immunogenicity, with a large multifunctional surface area. Researchers have thoroughly explored functionalized carbon-based nanomaterials to create biocompatible scaffolds and advance nanomedicine, highlighting their potential in medical fields [175].

### 5.3. Food and Agriculture

Nanotechnology could significantly transform modern farming methods [176]. Leaching, photolysis, drifting, hydrolysis, and microbial degradation are among the factors that cause most agrochemicals to be lost before reaching their intended targets [177]. Innovative techniques such as precision agriculture can help minimize these losses by applying agrochemicals more accurately and efficiently. With nanoparticles and nano capsules, pesticides and fertilizers can be delivered with a cost-effective and site-specific approach, reducing collateral damage. Goswami et al. discovered that various nanoparticles, including TiO_2_-NPs, Al_2_O_3_-NPs, SiO_2_-NPs, and ZnO-NPs, can inhibit infections caused by Sitophilus oryzae and the baculovirus B. mori virus in silkworms [178]. In addition, SiO_2_, CuO, ZnO, Mg, and Fe NPs have well-known applications as fertilizer developer [179]. Nanoparticles can also be used to detect and treat diseases in plants, as well as monitor soil conditions. Nanotechnology has the potential to revolutionize the agricultural industry, making it more sustainable and environmentally friendly. In the food industry, TiO_2_ and Ag NPs are widely used to detect volatile organic compounds despite toxicological concern [179].

### 5.4. Energy Generation, Storage, and Conversion

The use of metal nanoparticles (NPs) has become increasingly common in energy generation, conversion and storage devices like solar-cells, fuel cells, water-splitting systems, supercapacitors and lithium-ion batteries over the last few decades. Ag NPs and Cu NPs are frequently utilized in solar cells to transform photon energy into electrical power [180]. Green synthesized CuO nanoparticles derived from the Calotropis gigantea plant are utilized in dye-sensitized solar cells. The nanoparticles showed good photocatalytic activity and were found to be an efficient electron acceptor [181]. In fuel cells, chemicals such as hydrogen are converted into electrical energy, and water is released as a by-product. Yang et al. [182] investigated the impact of grain boundaries on glucose fuel cells using CuO nano bundles. They observed a faster glucose mass transfer due to the grain boundary dislocation surface terraces. Gold nanoparticles are utilized in fuel cell technology in various ways, such as the following: (i) they can speed up the oxygen reduction reaction (ORR) kinetics, thereby enhancing fuel cell efficiency; (ii) gold nanoparticles contribute to hydrogen purification by eliminating carbon monoxide impurities from hydrogen streams used in fuel cells; (iii) gold nanoparticles can generate H_2_O_2_, which can subsequently be used to produce electricity without light. Furthermore, gold nanoparticles can enhance conductance over platinum electrodes [183,184]. Cobalt oxide (Co_3_O_4_) nanoparticles are employed as high-capacity anodes in lithium-ion batteries. These nanoparticles are found to have excellent electrochemical properties, such as low charge transfer resistance and high specific surface area [185]. In addition, cobalt oxide (Co_3_O_4_) nanoparticles confined in mesoporous carbon showed promising performance in a supercapacitor [186]. Silicon nanoparticles (Si-NPs) demonstrate great potential as high-capacity anodes in lithium-ion batteries (LIBs). Silicon has a significantly higher theoretical capacity than graphite, with a gravimetric capacity of around 4200 mAh g^−1^, while graphite’s capacity is only 372 mAh g^−1^ [187].

### 5.5. Environmental Remediation

The process of nano-remediation involves utilizing nanomaterials to eliminate pollutants or contaminants from various environmental components, including soil, air, groundwater, sediment, and surface water. Nanomaterials are used to absorb, bind, or degrade pollutants or contaminants. Nano-remediation is an environmentally friendly and cost-effective alternative to traditional remediation methods. The use of CuO nanoparticles and nanocomposites in the treatment of water and wastewater was investigated by several researchers to remove toxic dyes and other harmful contaminants. CuO nanoparticles have been shown to effectively remove contaminants such as methylene blue, methyl orange, and rhodamine B from water [188,189,190]. Additionally, they can help in the degradation of phenolic compounds and heavy metals like lead and cadmium. These properties make CuO nanoparticles a promising solution for improving water quality. Infections spread by unsafe and dirty water have led to the deaths of millions of people, particularly children. A variety of nanomaterials have been described as potential wastewater treatment materials, such as polymeric nanoparticles, metal oxide nanoparticles, metal nanoparticles, carbon nano, and biopolymers. For example, incubation with Aspergillus niger broth containing Congo red demonstrated that silver nanoparticles decolorized over 85.8% within 24 h [191]. Nanometals and their related oxides, including MgO, TiO_2_, MnO_2_, ZnO, Fe_3_O_4_, and CdO, are frequently utilized to eliminate heavy metals, dyes, and ions from wastewater [192].

### 5.6. Catalysis

In catalysis and photocatalysis applications, bimetallic nanoparticles are instrumental because they accelerate the rate of reaction. They also enhance the optical system’s sensitivity and selectivity. Their unique structure allows for synergistic interactions between the two metals, which can create active sites with higher catalytic activity. This enhances the adsorption and activation of reactants, leading to faster reaction kinetics. Additionally, the electronic and geometric properties of bimetallic nanoparticles can be tuned to optimize the reaction pathway [63,107]. Bimetallic nanoparticles can expand the reaction scope of heterogeneous catalysis when combined with metal–organic framework (MOF) materials [193]. A few examples of heterogeneous catalysis are Pt–Au, Pt–Cu, Pt–Ni, Pt–Ru, Pd–Pt, Pd–Ag, Pd–Au, Au–Cu, Au–Rh, Au–Ag. As a catalyst, Ga nanoparticles are also widely used, for example, nanotube nanothermometers with Ga fillings [194], SnO_2_ nanowires and nanobelts formation [195], as well as highly aligned silica nanowires synthesis [196].

### 5.7. Sensor

Several applications of metal nanoparticles (NPs) have been identified as sensors, including biosensors, electrochemical sensors, and environmental monitoring. For instance, gold nanoparticles are widely used in biosensors for detecting biomarkers in medical diagnostics [62]. In biosensor applications, gold and gold-based NPs are useful for detecting small molecules, proteins, nucleic acids, tumor cells, metal ions, and several dozen other biological samples [63]. Gold-based nanoparticles enhance detection sensitivity due to their unique optical properties, such as surface plasmon resonance. When these nanoparticles interact with target molecules, they cause a shift in the plasmon resonance, which can be measured and analyzed. This change provides a highly sensitive and specific signal that facilitates the detection of various biological samples. Silver nanoparticles are employed in electrochemical sensors to enhance sensitivity in detecting heavy metals [197,198]. Additionally, zinc oxide nanoparticles are utilized in environmental monitoring sensors to efficiently detect pollutants in water and air [199]. Researchers are actively exploring the potential of combining metal nanoparticles with advanced materials like graphene to create highly sensitive and selective sensors [200]. These hybrid sensors could offer real-time monitoring capabilities and improved durability in harsh environments. Furthermore, the integration of nanotechnology with Internet of Things (IoT) platforms is expected to revolutionize remote sensing applications, enabling more comprehensive and accessible environmental and health monitoring systems.

**Figure 15:**
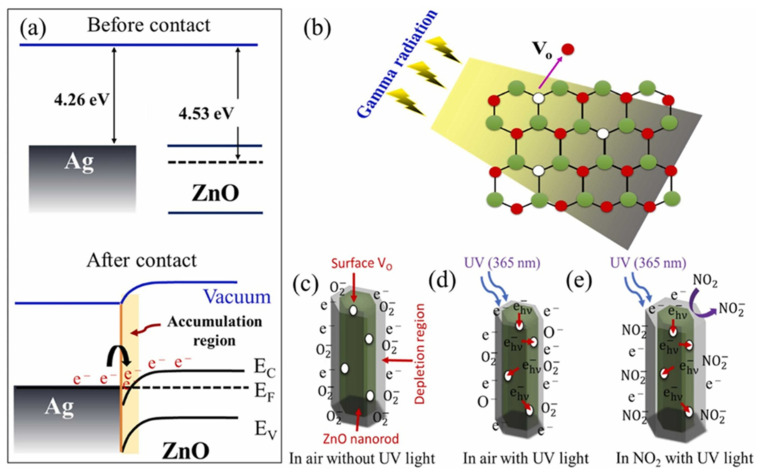
(**a**) Ag electrode and ZnO band alignment, (**b**) defect engineering via gamma irradiation, (**c**) gas sensing in air without UV light, (**d**) gas sensing in air with UV light, (**e**) gas sensing in NO_2_ with UV light, reproduced with permission from ref. [208], Elsevier.

Gas sensors application of metal oxide nanoparticles have been researched extensively in the last two decades and used successfully for the detection of several hazardous gases [201,202]. Detecting hazardous gases is crucial for maintaining environmental safety and ensuring industrial operations are secure. Gamma ray-modified metal oxide sensors have emerged as promising tools in this area, primarily due to their enhanced sensitivity and selectivity. The modification of metal oxides through gamma irradiation is particularly important as it alters both the surface properties and the electronic structure of the sensors. These changes facilitate better interactions between the sensors and gas molecules, improving detection precision and speed. Furthermore, irradiation can enhance the stability and durability of sensors, allowing them to function effectively in harsh conditions. In this section we will explore the latest advancements and applications of these sensors, emphasizing how irradiation-induced modifications propel their capabilities, leading to improved real-time gas monitoring and greater safety standards. Gamma increases sensor surface porosity, enhancing sensitivity to toxic gases. Studies have shown that modifying surface morphology with nanowires or nanoparticles significantly increases metal oxide sensor sensitivity, like in ZnO and SnO_2_ [203,204]. While chemical methods are well-studied, radiation-based physical methods remain underexplored. High-energy radiation induces surface changes like chemical methods, creating defects and structural modifications [205,206]. Gamma-irradiated metal oxide films have shown increased graininess and porosity. Abdullah F. Aldosary et al. highlighted enhanced ethane and ethylene sensitivity in gamma-irradiated ZnO sensors [207]. Similarly, Aswin Kumar Anbalagan et al. demonstrated improved NO_2_ sensing with gamma ray-created defects on ZnO nanorods at room temperature [208]. Figure 15 shows the band alignment and Ohmic junction between Ag and ZnO which are essential for gas sensing.

## 6. Conclusions and Future Direction

In conclusion, this review has comprehensively examined the utilization of electron beam and gamma radiation for the synthesis of nanomaterials. These radiation-based techniques offer significant advantages over traditional methods, including enhanced precision and control over nanoparticle properties, environmentally friendly synthesis pathways, and reduced energy consumption. The versatility of these methods is highlighted through the diverse applications discussed, ranging from the creation of high-purity metallic nanoparticles and semiconductor nanostructures to the fabrication of nanocomposites with tailored functionalities. We have detailed various synthesis procedures, highlighting the key parameters influencing nanoparticle characteristics such as size, shape, and distribution. The advantages of reduced chemical waste and improved sustainability are emphasized, aligning with the growing demand for green chemistry principles in materials science. While challenges concerning scalability and cost-effectiveness remain, the ongoing research efforts to optimize these methods showcase their considerable potential for future advancements in nanotechnology across various scientific and industrial applications. Future research should focus on addressing these challenges to further enhance the efficiency and scalability of radiation-based synthesis while investigating the long-term stability and environmental impact of resulting nanomaterials. The ultimate goal is to fully leverage the potential of this technology for widespread application in a sustainable and responsible manner.

Future research should prioritize enhancing the scalability and cost-effectiveness of radiation-based nanomaterial synthesis through optimized reactor designs and lower-energy radiation sources. Expanding material diversity by exploring new material classes and complex composites is crucial, alongside a deeper mechanistic understanding using advanced characterization and computational modeling to fine-tune nanoparticle properties. A thorough life-cycle assessment addressing environmental impact and safety concerns is necessary, while exploring synergistic combinations with other synthesis techniques can unlock novel material properties. Ultimately, translating laboratory-scale findings into real-world applications, including scalable manufacturing and device integration, is vital for fully realizing the potential of radiation-based nanomaterial synthesis.

## Figures and Tables

**Table 1:** Comparison of gamma rays and electron beam ionizing radiation on nanomaterials synthesis.

Characteristics	Gamma Rays Radiation Source	Electron Beam Radiation Source	Compounds	Gamma Irradiation Dose/Details	Electron Beam Irradiation Dose/Details	References
Power source	Radioactive nuclides (^60^Co)	Particle accelerators	Polypropylene	30 kGy, 60 kGy and 120 kGy	30 kGy, 60 kGy and 120 kGy	[163]
Equipment	Easy to operate and maintenance	Complicated to operate and maintenance	Low density polyethylene	100–2000 kGy	-	[164]
Processing time	Long	Short	LDPE	-	50–250 kGy	[165]
Dose rate	Low (10 kGy/h)	High (3 kGy)	Metallic Nanoparticles	Radiolytic reduction of HauCl_4_ in solution	Direct reduction of AgNO_3_ in solution	[59,72,134]
Shielding	Continuous operation requires	Can be switched on and off	Metal Oxide Nanoparticles	Irradiation of metal oxide precursor	Irradiation of metal oxide thin film	[86,87,88]
Penetration	Exponential attenuation	Finite range, depending on energy	Carbon-based nanomaterials	Reduction of graphene to graphene oxide	For precise patterning of graphene oxide	[127,140]

## Data Availability

No new data were created or analyzed in this study.
